# Evaluation of a Rapid Lateral Flow Assay for Coccidioidomycosis to Monitor Antibody Levels in Patients Using Fingerstick Capillary Blood

**DOI:** 10.3390/jof12050326

**Published:** 2026-04-29

**Authors:** Francisca J. Grill, Karen Pedersen, Kenta Reilly, Thomas E. Grys, Douglas F. Lake, Janis E. Blair

**Affiliations:** 1 School of Life Sciences, Arizona State University, Tempe, AZ 85287, USA; fgrill@asu.edu (F.J.G.); dlake1@asu.edu (D.F.L.); 2 Cactus Bio, LLC, Phoenix, AZ 85259, USA; 3 Division of Infectious Diseases, Mayo Clinic, Phoenix, AZ 85054, USA; 4 Department of Laboratory Medicine and Pathology, Mayo Clinic, Phoenix, AZ 85054, USA

**Keywords:** coccidioidomycosis, serology, point-of-care testing, lateral flow assay, antibody, Valley fever

## Abstract

The diagnosis of coccidioidomycosis is often achieved serologically by the detection of antibodies against fungal antigens. While several serologic tests are available for coccidioidomycosis, all of them are performed in a laboratory setting causing delays in diagnosis and therapeutic intervention. Point-of-care testing offers the ability to provide a shorter time to result by avoiding specimen send-out, minimizing processing steps, and employing expeditious immunochemical techniques. A preliminary trial of a rapid anti-coccidioidal antibody lateral flow assay (LFA) using fingerstick blood was performed on 22 patients with coccidioidomycosis at the point of care during outpatient clinic visits. Patients were tested longitudinally over the course of one year. An LFA reader was implemented to provide an objective result by quantifying the intensity of the test line band. There was close qualitative concordance observed between positive LFA results with send-out immunodiffusion (89.5%) and complement fixation (78.4%) standard of care clinical laboratory assays. Additionally, the relationship between LFA test line density values and traditional complement fixation antibody titers was assessed.

## 1. Introduction

Coccidioidomycosis is a fungal disease endemic to several regions in the southwestern United States as well as parts of Mexico, Central America, and South America [1,2]. Infection is caused by inhalation of spores from *Coccidioides* spp. (*immitis* and *posadasii*) which become airborne after both natural and human-related disturbances to the soil. Acute pulmonary coccidioidomycosis is common in the *Coccidioides*-endemic regions, estimated to be the cause of 15–20% of pneumonias within southern Arizona, but is symptomatically and radiographically indistinguishable from community-acquired pneumonia and other respiratory illnesses [3,4]. The gold standard for diagnosis is the microscopic observation of coccidioidal spherules in tissues or growth of *Coccidioides* spp. from patient specimens. Such specimens are often acquired using an invasive procedure, and the sensitivity of these methods is variable and depends on the type of sample obtained [2,5]. Thus, one composite reference standard is based on a diagnostic rubric that includes symptoms, radiographic findings, and serology (serum antibody testing) [6].

Antibody testing is typically performed first by enzyme immunoassay (EIA) in a clinical laboratory where isotype-specific secondary antibodies are used to detect anti-coccidioidal IgM and/or IgG. Specimens that test positive by EIA are subsequently assayed by a reference laboratory using immunodiffusion (ID) and complement fixation (CF) techniques [7]. ID and CF are laboratory-developed tests that employ traditional methods. Laboratories must source reagents from different suppliers, perform the assays manually, and interpret their subjective results. In particular, the complement fixation test is technically complex, and procedures are not standardized across laboratories. This results in variability of lab results, with reported antibody titers likely to be inconsistent if sent to separate laboratories, though this has not been characterized in recent years [8,9,10]. The turnaround time for ID and CF results varies but is listed as 2–7 days from time of specimen receipt for high-volume reference laboratories with highly trained personnel [11,12]. This extended turnaround time may discourage healthcare providers in emergency departments and urgent care centers from ordering *Coccidioides* serology [13]. This situation emphasizes a need for an accurate and rapid test that can be conducted at site of initial clinical presentation, whether in an urgent care facility, emergency department, or the office of a primary care medical provider.

Our group developed and previously published results of a lateral flow assay (LFA) that can detect anti-coccidioidal antibodies in serum and plasma in 10 min [14]. Since a 10-min test can be performed within the time frame of an office visit, we evaluated the utility of this assay in a point-of-care setting. Capillary blood from a fingerstick was used to detect and monitor antibodies in patients with coccidioidomycosis, and the LFA results were compared to standard of care ID and CF results from a reference laboratory. In prior studies testing serum and plasma specimens, a positive correlation between test line density unit values and CF antibody titers has been observed [14,15]. In this study, an LFA reader was similarly implemented to determine if the LFA was also semi-quantitative when using fingerstick blood.

## 2. Methods

The study protocol was approved by the Institutional Review Board (IRB) of Mayo Clinic, and written informed consent was obtained electronically or in person from all participants [IRB protocol number 23-010505]. Immunocompetent adult patients were considered for inclusion if they had proven or probable pulmonary coccidioidomycosis with a detectable complement fixation titer within three months from study enrollment. Since enrolled patients would be monitored serologically every three months as part of standard of care, an LFA would be performed concurrently within one to two weeks of send-out testing.

**Figure 1:**
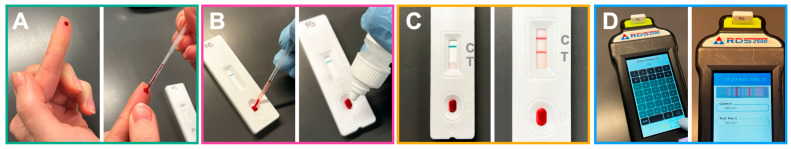
Illustration of LFA fingerstick application. (**A**). Finger is pricked with a spring-loaded 28G lancet and a 10 µL marked microcapillary pipette is used to sample 10 µL of capillary blood. (**B**) The collected blood is immediately dispensed into the sample port of the LFA cassette followed by 3 drops of chase buffer. (**C**) A 10 min timer is started when the sample enters the viewing window. If the sample is antibody-positive, a red line appears at the test line (T), as shown in the image. The absence of a red line at the test line (T) indicates a negative result (not pictured). (**D**) After 10 min, the LFA cassette is inserted into a programmed LFA reader that quantifies the intensity of the control line (C) and test line (T) to provide an objective, semi-quantitative result in the form of test line density units.

The LFA was performed as previously described [14] with preliminary steps added for the collection of fingerstick blood. Two clinic personnel underwent a 30 min training session on the methodology of the LFA process, and written instructions were provided to the testers for reference. Briefly, a 28-gauge pressure-activated lancet (Carelife USA Inc., Duluth, GA, USA) was used to obtain a blood sample by pricking the fingertip of a consented participant. Blood was sampled using a microcapillary pipette marked with a 10-microliter volume (KH Medical, Dongtai, Jiangsu, CN) and subsequently dispensed into the sample port of the LFA cassette followed by three drops of chase buffer. After 10 min, results were interpreted visually, and test line density was measured using a programmed RDS-2500 LFA reader (Detekt Biomedical, Austin, TX, USA). A test line density unit value ≥ 40,000 was considered a positive result, and a value < 40,000 was recorded as negative. Since this was the first investigation of the LFA with whole blood sample types, this cutoff was chosen based on prior testing performed with serum on this iteration of the LFA which yielded a cutoff value of 30,000 density units [16]. However, the cutoff value was increased to 40,000 density units with the assumption that fingerstick capillary blood may result in an increase in background noise due to the increased likelihood of hemolysis. This cutoff value was determined prior to study initiation. Figure 1 depicts a summary of the steps in performing the lateral flow assay. The test reported in the current study was a research prototype and was not commercially available at the time of testing.

The LFA results from fingerstick blood were compared to CF and ID test results performed by a reference laboratory (Mayo Clinic Laboratories, Rochester, MN, USA) using serum collected by phlebotomy as part of standard of care testing. Because only patients with a positive CF titer were enrolled (i.e., patients with serologically positive coccidioidomycosis), only positive percent agreements between the LFA and reference assays (ID and CF) were calculated.

## 3. Results

An initial LFA was performed on fingerstick blood from 34 participants enrolled in the study. Seven participants were dropped from the study due to a screen failure (prior positive CF titer, but negative recent titer). An additional participant was excluded because the reference laboratory results were collected more than two weeks after the LFA was performed, and four participants withdrew from the study after collection of the first time point. The remaining 22 participants were longitudinally monitored for anti-coccidioidal antibodies over a one-year period. A total of 54 LFA results were recorded using the LFA reader. However, five test results were excluded due to human error during the reading process. With these exclusions, of the 22 enrolled participants, there were 3 participants with one LFA result recorded, 11 with two results recorded, and 8 with three results recorded over the study period (December 2023 to December 2024). No patients had more than three LFA results recorded.

For the duration of the study, 3 of the 22 patients remained seropositive by ID, 1 patient converted to seropositive by ID, 1 patient initially seropositive became seronegative then returned to seropositive by ID, 1 patient initially seronegative became seropositive then returned to seronegative by ID, 10 patients became seronegative and remained seronegative by ID, and 6 patients were never seropositive by ID despite being seropositive by CF. A total of 19 positive ID results were reported that had an accompanying LFA result. The LFA agreed with ID positivity for 17/19 tests (89.5%).

For the duration of the study, 13 of the 22 patients remained seropositive by complement fixation, 8 patients converted from seropositive to seronegative and remained seronegative by CF, and 1 patient who was initially seropositive became seronegative then again became seropositive by CF. A total of 37 positive CF results were reported that had an accompanying LFA result. The LFA agreed with CF positivity for 29/37 tests (78.4%), while ID results agreed with CF positivity for only 22/42 tests (52.4%).

**Figure 2:**
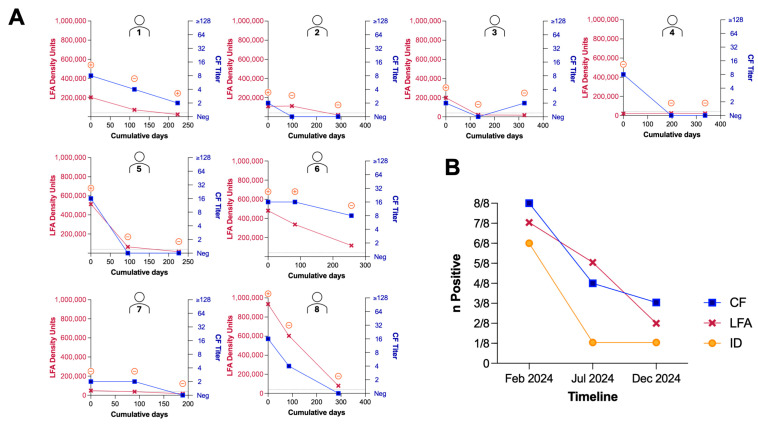
Comparison of CF antibody titer, ID reactivity, and LFA test line density units over study duration for 8 patients with three timepoints. (**A**) LFA test line density units are graphed on the left Y axis (red) and CF antibody titers are graphed on the right Y axis (blue). ID results are shown in an orange bubble above each timepoint, with ⊕ indicating a positive ID result and ⊖ indicating a negative ID result. CF, ID, and LFA tests were run singularly without replicates. (**B**) Number of patients positive by CF (blue), LFA (red), and ID (orange) over the study duration for participants with three timepoints recorded.

We also wanted to evaluate the utility of the LFA to monitor relative antibody levels of patients in real-time longitudinally. An LFA reader was implemented to provide a quantitative value of the test line density to determine if there was a correlation between the intensity of the test line and the reported CF antibody titer. The ID, CF, and LFA results for each of the eight patients that had three LFA results recorded longitudinally are shown in Figure 2A. The total number of positive tests for the eight patients with three time points over the duration of the study is shown in Figure 2B. In general, it appeared that patients first became seronegative by ID, then negative by CF and the LFA (Figure 2B). The median interval between LFA testing visits for this subset of eight patients was 117 days (IQR 97–177 days).

## 4. Discussion

While anti-coccidioidal antibody testing is not confirmatory for diagnosis on its own, it is heavily relied upon within *Coccidioides*-endemic areas to support a diagnosis of coccidioidomycosis in the context of compatible clinical and radiographic findings. Both complement fixation (CF) and immunodiffusion (ID) assays are typically performed as send-out testing to reference laboratories, adding days to the testing process, delaying a potential diagnosis, and in some cases, discouraging testing by emergency department physicians [13]. Enzyme immunoassays (EIAs) and a commercially available LFA, sōna, offer more rapid turnaround times than CF and ID but are still not practical in the context of emergency departments and urgent care facilities due to the technical steps required which are better suited to be performed in a laboratory environment (e.g., need for phlebotomy and centrifugation to obtain serum followed by a two-step dilution of patient specimen). Currently, there are no tests for coccidioidomycosis that are compatible with whole blood as a specimen type. This study investigated the utility of a recently developed lateral flow assay [14] to be utilized with peripheral blood acquired through a minimally invasive fingerstick that provides a result in 10 min.

The LFA presented here demonstrated positive percent agreements of 89.5% and 78.4% with ID and CF tests, respectively. Because positive percent agreement is constrained by the performance of the reference standard, these metrics may not fully capture the performance of a new test. Interestingly, ID testing showed only a 52.4% positive percent agreement with CF, highlighting the discordance that already exists between current standard of care testing methods and underscoring the difficulty of evaluating a new assay. Part of this low agreement could be due to differences in the analytical sensitivity of ID and CF tests. Since it is unclear which test is more sensitive, performing ID and CF concurrently has been recommended [17]. The analytical sensitivity of the LFA using whole blood is unknown. Another cause of discordance between ID and CF testing could be that the tests are complex to perform and results are subject to interpretation by the laboratory staff. While the LFA presented here is much simpler for laboratory staff to use and interpret qualitatively as positive or negative, errors were made by clinical personnel when using the reader, despite an educational training session, potentially raising concerns about incorporating the reader at the bedside or point of care. The rationale for using an LFA reader was to eliminate subjective user interpretation of the result as well as provide a semi-quantitative output with a numerical cutoff value to determine a positive result. However, incorrect insertion of the LFA cassette into the reader led to the invalidity (no recorded test line density) of 5/54 (9.3%) of the tests performed. Technical safeguards and design optimization could mitigate such errors in future iterations of the test.

The current study took place in an outpatient clinic with subjects who carried a diagnosis of coccidioidomycosis with proven or probable disease who were being monitored every three to four months. We observed a correlation between LFA test line intensity and CF antibody titers for eight subjects monitored at three timepoints (Figure 2A). While the ability of the LFA to track relative antibody levels is promising and may be useful in low-resource settings, the authors of this paper postulate that the best utility of this point-of-care qualitative LFA would be at initial presentation in urgent care facilities or emergency departments. In these healthcare facilities, turnaround time is essential to aid in the differential diagnosis of coccidioidomycosis among other respiratory infections. Since the patients enrolled were those with seropositive coccidioidomycosis, the ability of the LFA to screen for the initial diagnosis of coccidioidomycosis during acute presentation was not evaluated. Gold nanoparticle-based LFA formats have been reported to exhibit lower sensitivities than enzyme immunoassays [18]; therefore, a subsequent study directly comparing LFA and EIA performance at the time of initial presentation would be valuable to assess if any tradeoff in analytical sensitivity for diagnostic speed exists. Another limitation to this study is that the patients enrolled were immunocompetent, and performance of the LFA in immunocompromised patients who may not mount an antibody response detectable by ID or CF serologic tests was not assessed. Future studies in both immunocompetent and immunocompromised individuals in the setting of emergency departments or urgent care clinics will be important to demonstrate the point-of-care utility of this LFA, as well as to determine its specificity in the context non-coccidioidal community-acquired pneumonias. The early identification of coccidioidomycosis could theoretically reduce unnecessary antibiotics for patients and have a positive impact on antimicrobial stewardship.

## Data Availability

The data presented in this study is contained within the article. Further inquiries can be directed to the corresponding author.
